# Mitochondrial dysfunction and impaired DNA damage repair through PICT1 dysregulation in alveolar type II cells in emphysema

**DOI:** 10.1186/s12964-024-01896-0

**Published:** 2024-11-22

**Authors:** Hannah Simborio, Hassan Hayek, Beata Kosmider, John W. Elrod, Sudhir Bolla, Nathaniel Marchetti, Gerard J. Criner, Karim Bahmed

**Affiliations:** 1 Center for Inflammation and Lung Research, Lewis Katz School of Medicine at Temple University, 3500 N. Broad Street, Philadelphia, PA 19140 USA; 2 Department of Microbiology, Immunology, and Inflammation, Lewis Katz School of Medicine at Temple University, Philadelphia, PA 19140 USA; 3 Aging & Cardiovascular Discovery Center, Lewis Katz School of Medicine at Temple University, Philadelphia, PA 19140 USA; 4 Department of Thoracic Medicine and Surgery, Lewis Katz School of Medicine at Temple University, Philadelphia, PA 19140 USA

**Keywords:** COPD, ATII cells, Mitochondrial respiration, Cell cycle, Apoptosis, Double strand breaks, OXPHOS, Oxidative stress

## Abstract

**Background:**

Alveolar type II (ATII) cells have a stem cell potential in the adult lung and repair the epithelium after injury induced by harmful factors. Their damage contributes to emphysema development, characterized by alveolar wall destruction. Cigarette smoke is the main risk factor for this disease development.

**Methods:**

ATII cells were obtained from control non-smoker and smoker organ donors and emphysema patients. Isolated cells were used to study the role of PICT1 in this disease. Also, a cigarette smoke-induced murine model of emphysema was applied to define its function in disease progression further.

**Results:**

Decreased PICT1 expression was observed in human and murine ATII cells in emphysema. PICT1 was immunoprecipitated, followed by mass spectrometry analysis. We identified MRE11, which is involved in DNA damage repair, as its novel interactor. PICT1 and MRE11 protein levels were decreased in ATII cells in this disease. Moreover, cells with PICT1 deletion were exposed to cigarette smoke extract. This treatment induced cellular and mitochondrial ROS, cell cycle arrest, nuclear and mitochondrial DNA damage, decreased mitochondrial respiration, and impaired DNA damage repair.

**Conclusions:**

This study indicates that PICT1 dysfunction can negatively affect genome stability and mitochondrial activity in ATII cells, contributing to emphysema development. Targeting PICT1 can lead to novel therapeutic approaches for this disease.

**Supplementary Information:**

The online version contains supplementary material available at 10.1186/s12964-024-01896-0.

## Background

Abnormal inflammatory responses of the lungs to noxious particles or gases, mainly cigarette smoke, are associated with chronic obstructive pulmonary disease (COPD) development [1, 2]. Emphysema is a form of COPD and pathologically is characterized by permanent airflow restriction resulting from a unique pattern of alveolar wall destruction, enlargement of alveolar airspace, and loss of lung structure and elasticity [3–6]. Alveolar type II (ATII) cells play a major role in lung homeostasis and serve as a stem cell pool [7, 8]. They are abundant in mitochondria, which play a critical role in cell metabolism, and their dysfunction can contribute to respiratory diseases [7, 9].

Increased oxidative stress is one of the most consistently observed alterations in COPD [1]. Also, cigarette smoke induces the recruitment of inflammatory cells, which then release proteolytic enzymes, causing the degradation of the lung matrix and the death of structural cells. Disruption of the balance between apoptosis and replenishment of these cells in the lung can contribute to the destruction of lung tissue, leading to emphysema. We have previously reported that ATII cells isolated from emphysema patients exhibit mitochondrial (mt) DNA damage and dysfunction and correlated with increased ROS levels [8, 10]. MRE11 possesses DNA nuclease activities, is involved in DNA damage repair, and forms the MRE11-RAD50-NBS1 (MRN) complex, which binds to double-strand DNA breaks [11].

Protein interacting with carboxyl terminus-1 (PICT1, also known as GLTSCR2 or NOP53) is a multifunctional protein that enhances mitochondrial function for the maintenance of oxygen consumption, consistent with a pivotal role in the control of cellular respiration [12]. It responds to mitochondrial stress and regulates cellular proliferation and metabolism via the transcription factor MYC. The *Pict1* loss impairs the survival of mouse embryos and embryonic stem cells, which proves that it is essential for preimplantation embryogenesis [13].

To our knowledge, the role of PICT1 in ATII cells or emphysema is unstudied. Here, we investigated its function in cigarette smoke-induced emphysema using human primary ATII cells and a mouse model. To our knowledge, there are only several reports on the role of PICT1 in the lung cells [14–16], and our study is the first to indicate the functional role of PICT1 and MRE11 interaction. Our data highlight the impact of PICT1 deficiency on emphysema development. Its stabilization can lead to potential therapeutic approaches in this disease.

## Methods

### Human ATII cell isolation

Lungs were obtained from 20 non-smoker organ donors (11 females and 9 males, age 63.55 ± 3.16), 16 smoker organ donors (10 females and 6 males, age 58.19 ± 2.19), and 17 patients with emphysema (6 females and 11 males, age 62.56 ± 1.46). ATII cells were isolated, as we reported [17]. The IRB at Temple University approved this research. Informed written consent was obtained to use lungs for research.

### Mass spectrometry analysis

We immunoprecipitated PICT1 in human lung tissue to identify its interactors. Mass spectrometry analysis with a standard protein identification strategy was used, as we previously described [18]. Trypsin digestion was applied to analyze MS/MS spectra (tandem mass spectrometry) generated from the LC–MS/MS runs (liquid chromatography with tandem mass spectrometry).

### Mice exposure to cigarette smoke

Male and female wild-type C57BL/6 mice (Jackson Laboratories) of 4–5 weeks of age were used in experiments. Cigarette smoke was generated from research cigarettes 3R4F (Kentucky Tobacco Research & Development Center). Mice were exposed to 300 mg/m^3^ total suspended particles (TSP) for 2 h for 5 days per week for 3 weeks or 8 months using a Teague TE-10 smoking system (Teague Enterprises). The IACUC at Temple University approved all experimental procedures.

### Murine lung histology and micro-CT

Murine lung sections were stained using hematoxylin (Ricca) and eosin solutions (Astral Diagnostics). Images were captured with brightfield microscopy (Zeiss). Minimum, maximum, and mean alveolar diameters were measured using Image Pro v10.0.14 (Media Cybernetics) [19]. Also, lung tissue was analyzed using a micro-CT scanner (Bruker). Standard alcohol fixation and hexamethyldisilazane (Sigma) were applied as described [20]. Analysis was performed using Bruker software per the manufacturer’s recommendations.

### Chest CT scans

CT scans were performed using standard procedures, and 3D SLICER software was applied for computerized image analysis [21]. The study was conducted in accordance with the Declaration of Helsinki and was approved by IRB at Partners Healthcare and the Committee for the Protection of Human Subjects at Temple University.

### PICT1 deletion

A549 cells, a model of ATII cells, were cultured and maintained in DMEM (GE Healthcare), supplemented with 10% FBS (GE Healthcare), 100 μg/ml streptomycin, and 100 U/ml penicillin (Thermo Fisher Scientific). PICT1 knockout A549 cell line was generated using PICT-1 CRISPR plasmid (Santa Cruz Biotechnology) and CRISPR-Cas9 technology, as we previously described [18]. Mouse lung epithelial MLE15 cells were cultured and maintained in HITES medium formulated as follows: DMEM:Ham’s F12 (GE Healthcare, Gibco) with 50:50 mix, 0.005 mg/ml insulin, 0.01 mg/ml transferrin, 20 nM sodium selenite, 10 nM hydrocortisone (all from Lonza), 2% fetal bovine serum (GE Healthcare), 2 mM L-Glutamine, 100 μg/ml streptomycin and 100 U/ml penicillin (all from Thermo Fisher Scientific). MLE15 cells were treated with 100 nM non-target (NT) siRNA or PICT1 siRNA (Santa Cruz Biotechnology) using RNAiMAX transfection reagent (Thermo Fisher Scientific) for 48 h, according to the manufacturer’s instructions. Cells with PICT1 knockdown were confirmed using Western blotting.

### Comet assay

The OxiSelect Comet Assay (Cell Biolabs) was used to measure DNA damage. Individual nuclei of wild-type and PICT1-deficient A549 cells treated with cigarette smoke extract were analyzed. The manufacturer’s instructions were followed.

### Western blotting

Western blotting was performed to analyze protein levels. Briefly, membranes were incubated with primary antibodies PICT1, MRE11, 53BP1, LIGASE IV, KU80, VDAC, and β-actin (Santa Cruz Biotechnology), LAMIN B (Proteintech), and TRIM22 (Bioss). HRP-conjugated donkey anti-rabbit or anti-mouse IgG (Jackson ImmunoResearch) were applied. A chemiluminescent detection agent (Millipore) was used. The quantification of band intensity was analyzed using Image J (NIH) software.

### RT-PCR

Cells and lung tissue were homogenized in RLT buffer (Qiagen). RNA was isolated using RNA Isolation Kit (Qiagen), according to the manufacturer’s recommendations. RNAs were reverse transcribed to cDNA, and RT-PCR was performed. Human and murine primer sequences are shown in Supplementary Table 1 and Supplementary Table 2. Reactions were carried out using a StepOnePlus Real-Time PCR System (Applied Biosystems). Data were analyzed using the ΔΔCt method.

### Immunohistofluorescence

Paraffin-embedded human and murine lung tissue sections were incubated overnight with antibodies: SP-C (Santa Cruz Biotechnology and Abclonal), PICT1 (Santa Cruz Biotechnology), MRE11, or TOM20 (Santa Cruz Biotechnology and Proteintech). Secondary antibodies, Alexa Fluor 594, Alexa Fluor 488, and Alexa Fluor 647 (Invitrogen), were used for 1 h. Control IgG staining was performed. A MOM staining kit (Vector Laboratories) was used for murine tissue. Nuclei were labeled using a Fluoroshield Mounting Medium containing DAPI (Abcam). Images were captured by confocal laser-scanning microscopy (Zeiss), and fluorescence intensity was quantified with ImageJ (NIH) software.

### Mitochondrial function

Mitochondrial DNA (mtDNA) amount, mtDNA damage, and common deletion were determined by qPCR as we previously described [10]. MitoSOX Red (Thermo Fisher Scientific) and Cell Mito Stress Test (Agilent) were used according to the manufacturer’s instructions. The mitochondrial network was also analyzed using ImageJ (NIH) software [22].

### Flow cytometry analysis

Wild-type and PICT1-deficient A549 cells were treated with 0, 125, 250, and 500 µM hydroxyurea (Sigma) or 20% cigarette smoke extract for 24 h and analyzed using Alexa Fluor 488 Annexin V/Dead Cell Apoptosis kit (Thermo Fisher Scientific) per manufacturer’s instructions. ROS generation was determined using a DCF-DA probe (Thermo Fisher Scientific). The cell cycle was analyzed using 1 μg/ml propidium iodide (PI) (Thermo Fisher Scientific).

### Statistics

The obtained results were analyzed using GraphPad-Prism and expressed as means ± SEM from at least three experiments. One-way ANOVA and *t*-test were used. The results with *p* < 0.05 were considered statistically significant.

## Results

### Decreased PICT1 protein levels in emphysema

**Fig. 1:**
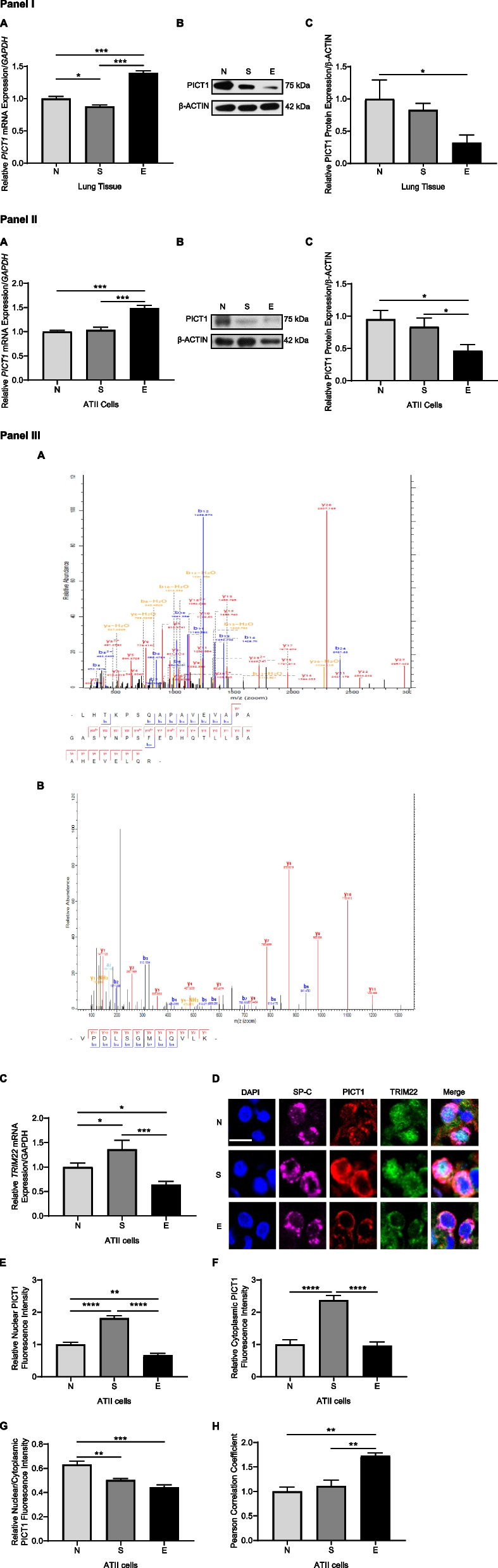
Decreased PICT1 protein expression in ATII cells in emphysema patients. Lung tissue and ATII cells were obtained from control non-smoker (N) and smoker (S) organ donors and emphysema patients (E). Panel I: A—*PICT1* mRNA levels in lung tissue by RT-PCR. B—Representative Western blot images of PICT1 expression in lung tissue. C—Quantification of protein expression normalized to β-actin is shown. Panel II: A—*PICT1* mRNA levels in ATII cells by RT-PCR. B—Representative Western blot images of PICT1 expression in ATII cells. C—Quantification of protein expression. Panel III: A – PICT1 was immunoprecipitated in lung tissue, followed by mass spectrometry analysis. Representative PICT1 (**A**) and TRIM22 spectrum (**B**) are shown. **C** *TRIM22* mRNA expression in ATII cells by RT-PCR. **D** ATII cells were stained in lung tissue sections using SP-C (magenta), PICT1 (red), and TRIM22 (green) antibodies and DAPI (blue) followed by analysis by immunofluorescence (scale bar—5 μm). PICT1 fluorescence intensity in the nucleus (**E**) and cytoplasm (**F**) was quantified. **G** The ratio of nuclear to cytoplasmic PICT1 fluorescence intensity. **H** Pearson’s correlation coefficient for PICT1 and MRE11 fluorescence co-localization in ATII cells. Data are shown as means ± SEM (*N* = 3—14 lungs per group). *p < 0.05, **p < 0.01, ***p < 0.001, ****p < 0.0001

*PICT1* mRNA levels were increased in lung tissue obtained from emphysema patients compared to non-smokers and smokers (Fig. 1, Panel I, A). Reduced PICT1 protein expression was found in this disease in comparison with non-smokers (Fig. 1, Panel I, B, C). We also isolated ATII cells from lungs obtained from non-smokers, smokers, and individuals with emphysema. The highest *PICT1* mRNA level was detected in ATII cells in this disease (Fig. 1, Panel II, A). However, PICT1 protein expression was the lowest in this group (Fig. 1, Panel II, B, C), which suggests posttranslational regulation.

PICT1 was immunoprecipitated in lung tissue obtained from non-smokers, smokers, and emphysema, followed by mass spectrometry analysis. We identified its interaction with TRIM22, a class of E3 ubiquitin ligases (Fig. 1, Panel III, A, B). *TRIM22* mRNA levels were the lowest in ATII cells in emphysema (Fig. 1, Panel III, C). We also analyzed PICT1 and TRIM22 expression in ATII cells (Fig. 1, Panel III, D). Decreased PICT1 levels were found in the cytoplasm and nucleus in emphysema patients compared to smokers (Fig. 1, Panel III, E, F). The lowest PICT1 nuclear/cytoplasm ratio and the highest PICT1 and TRIM22 co-localization were detected in ATII cells in this disease (Fig. 1, Panel III, G, H).

*PICT1* mRNA levels increased in lung tissue in severe emphysema, and we didn’t detect significant differences in the protein expression between analyzed groups (Supplementary Fig. 1, Panel I, A, B, C). There was no difference between PICT1 levels in ATII cells obtained from mild and severe emphysema (Supplementary Fig. 1, Panel II, A, B). We also analyzed PICT1 expression in cytosolic, nuclear, and mitochondrial fractions obtained from lung tissue from non-smokers, smokers, and patients with this disease. There were no significant changes between the analyzed groups (Supplementary Figs. 2A-F). Our results suggest that decreased PICT1 expression may contribute to emphysema pathophysiology.

### PICT1 and MRE11 interaction 

**Fig. 2:**
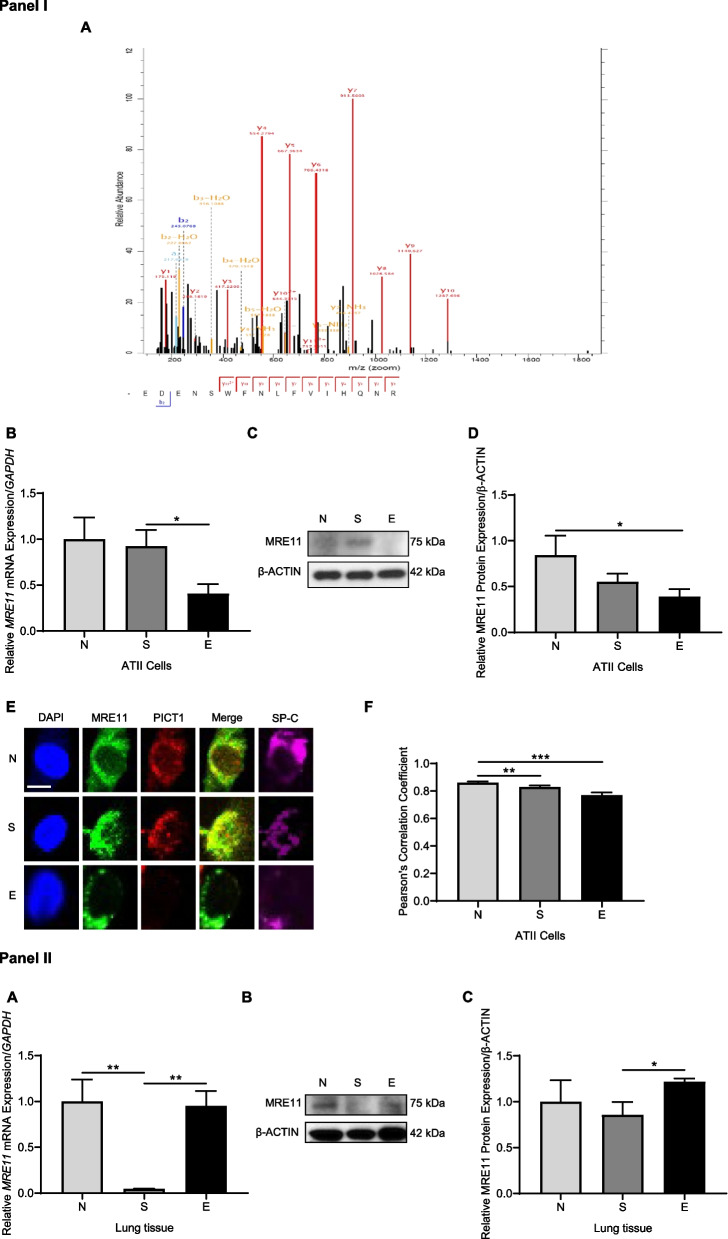
Reduced MRE11 expression in ATII cells in emphysema. Lung tissue and ATII cells were obtained from non-smokers (NS), smokers (S), and emphysema patients (E). Panel I: PICT1 was immunoprecipitated in lung tissue, followed by mass spectrometry analysis. **A** Representative MRE11 spectrum is shown, which was identified as a PICT1 interactor. **B** *MRE11* mRNA levels in ATII cells by RT-PCR. **C** Representative Western blot images of MRE11 expression in ATII cells. **D** Quantification of protein expression normalized to β-actin is shown. **E** MRE11 (green) and PICT1 (red) antibodies and DAPI (blue) were applied using immunofluorescence. ATII cells were identified in lung tissue sections using SP-C (magenta, scale bar 5 μm). **F** Pearson’s correlation coefficient for PICT1 and MRE11 fluorescence co-localization in ATII cells is shown. Panel II: A *MRE11* mRNA levels in lung tissue by RT-PCR. B Representative Western blot image of MRE11 expression in lung tissue. C—Quantification of protein expression is shown. Data are shown as means ± SEM (*N* = 3 – 19 lungs per group). *p < 0.05, **p < 0.01, ***p < 0.001

PICT1 was immunoprecipitated, followed by mass spectrometry analysis. We identified MRE11 as a novel PICT1 interactor in the lung (Fig. 2, Panel I, A). Our results indicate the lowest *MRE11* mRNA and protein levels in ATII cells in individuals with emphysema (Fig. 2, Panel I, B, C, D). We detected decreased PICT1 and MRE11 co-localization in ATII cells in smokers and this disease compared to non-smokers (Fig. 2, Panel I, E, F). Reduced *MRE11* expression was detected in lung tissues in smokers compared to non-smokers or emphysema patients (Fig. 2, Panel II, A). MRE11 protein levels were higher in this disease than in smokers (Fig. 2, Panel II, B, C). Many cell types are present in lung tissue, which can explain the obtained results. We identified PICT1 and MRE11 interaction and decreased MRE11 levels in ATII cells in emphysema.

### Reduced PICT1 levels in ATII cells in murine emphysema

**Fig. 3:**
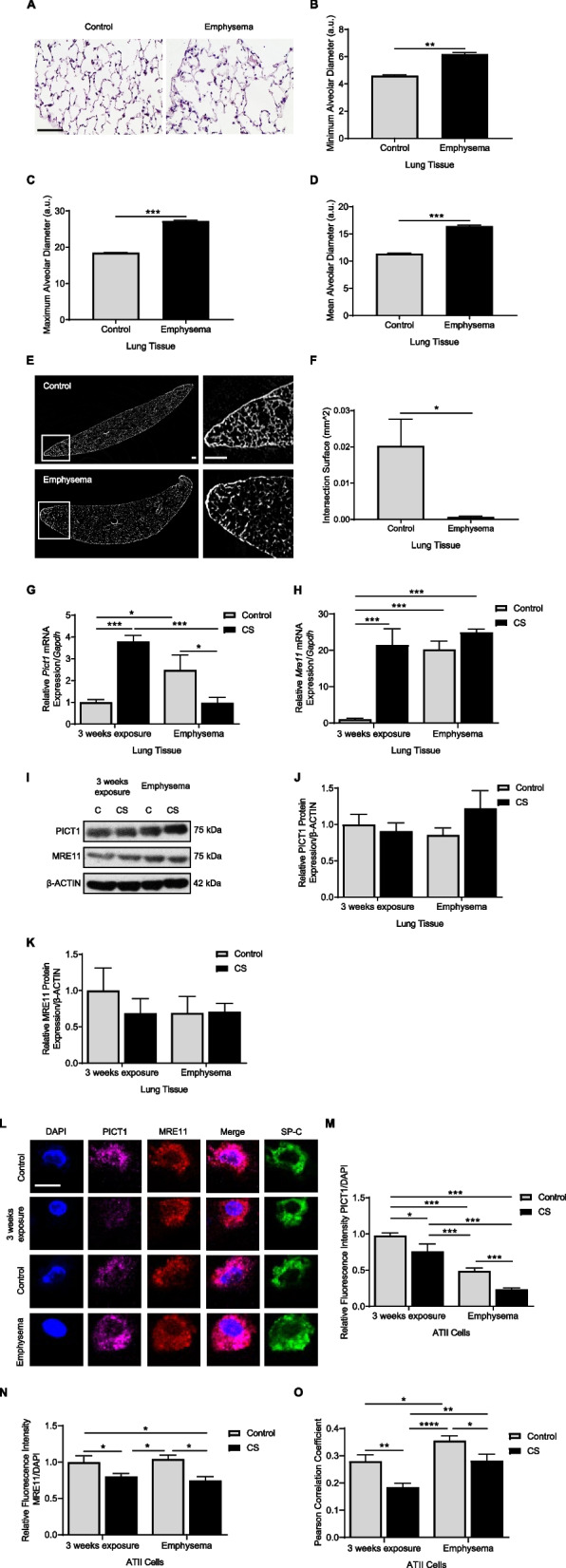
Decreased MRE11 protein levels in ATII cells in a murine model of emphysema. Wild-type mice were exposed to cigarette smoke for 8 months, as described in the Methods section, to induce emphysema. **A** Hematoxylin and eosin staining in murine lung tissue (scale bar 50 μm). Minimum (**B**), maximum (**C**), and mean (**D**) alveolar diameters were measured in lung tissue sections. **E** Representative micro-CT of the murine lung (scale bar-100 μm). **F** The intersection surface was quantified using micro-CT images. **G** *Pict1* and **H** *Mre11* mRNA levels were evaluated in lung tissue by RT-PCR. **I** Representative Western blotting images of PICT1 and MRE11 expression in lung tissue. PICT1 (**J**) and MRE11 expression (**K**) are quantified. **L** ATII cells in lung tissue sections were identified using SP-C (green). PICT1 (magenta), and MRE11 (red) antibodies, and DAPI (blue) by immunofluorescence (scale bar—5 μm). Quantification of PICT1 (**M**) and MRE11 (**N**) fluorescence intensity in ATII cells is shown. **O** Pearson’s correlation coefficient for PICT1 and MRE11 fluorescence co-localization. Data are shown as means ± SEM (*N* = 3 – 8 mice per group). *p* < 0.05, **p < 0.01, ***p < 0.001, ****p < 0.0001

We used a mouse model of cigarette smoke-induced emphysema to determine the role of PICT1. Significantly increased minimum, maximum, and mean alveolar diameters were observed in these mice compared to controls (Figs. 3A, B, C, D). We also applied micro-CT and found a significantly decreased lung parenchyma intersection surface in emphysema (Figs. 3E, F).

Next, we determined *Pict1* and *Mre11* mRNA and protein levels using lung tissue obtained from mice exposed to cigarette smoke for 3 weeks and 8 months, where the latter induced emphysema. Our results showed increased *Pict1* mRNA expression after short exposure to cigarette smoke and a decrease in emphysema compared to controls (Fig. 3G). *Mre11* levels were higher in mice exposed to cigarette smoke and in older control mice (Fig. 3H). We did not see significant differences in PICT1 and MRE11 levels in lung tissue after 3 weeks of exposure to cigarette smoke and in emphysema (Figs. 3I, J, K). Also, we analyzed PICT1 levels in cytoplasmic and nuclear fractions from lung tissue obtained from mice with cigarette smoke-induced emphysema. We found its decreased expression in the cytoplasm (Supplementary Figs. 3A, B, C, D).

We found a progressive decline in PICT1 expression in analyzed conditions, and its lowest level was detected in murine ATII cells in emphysema (Figs. 3L, M). Moreover, MRE11 levels were decreased after mice exposure to cigarette smoke for 3 weeks and in emphysema (Fig. 3N). A similar correlation was observed for the co-localization of PICT1 and MRE11 in ATII cells (Fig. 3O). Together, our data suggest decreased PICT1 and MRE11 expression in ATII cells in the murine model of cigarette smoke-induced emphysema.

### PICT1 deficiency sensitizes cells to DNA damage

**Fig. 4:**
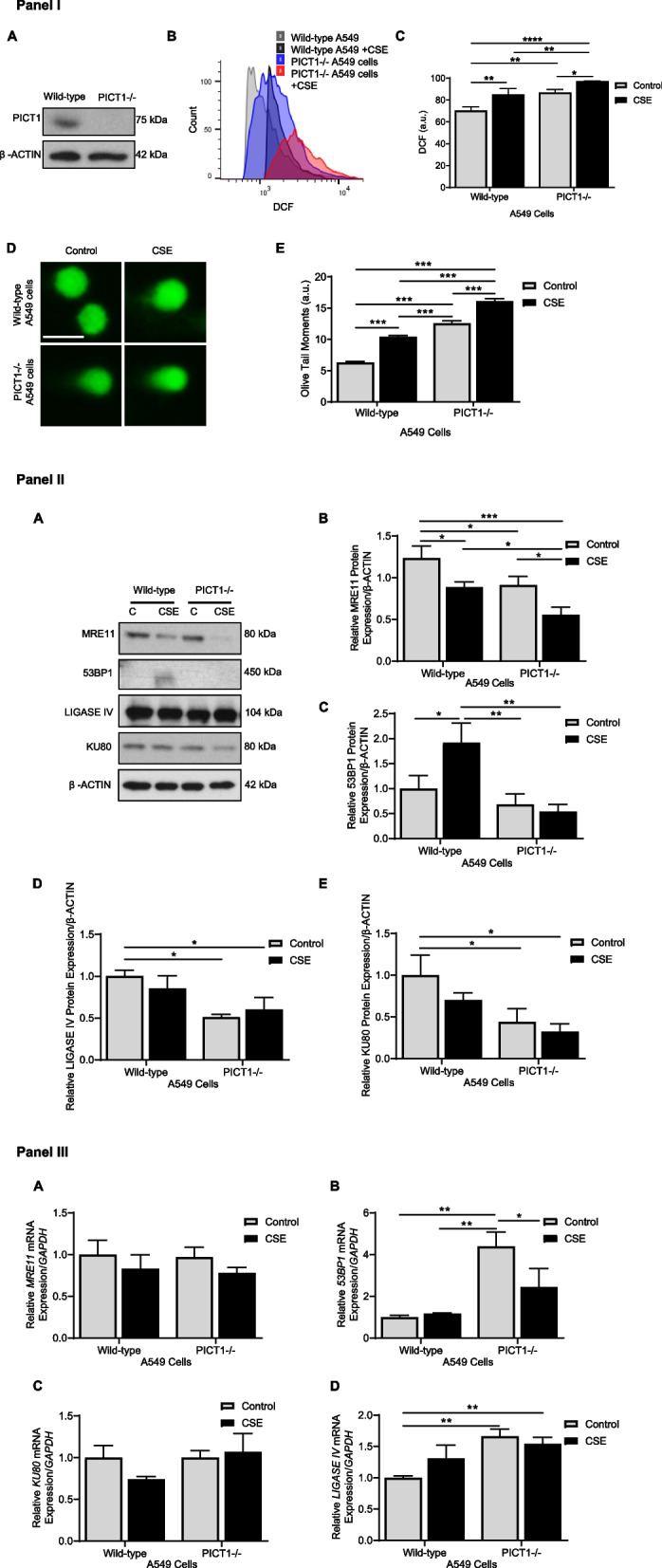
PICT1 deletion impairs DNA damage repair in A549 cells. Panel I: CRISPR-Cas9 strategy was used to generate cells with PICT1 deletion as described in the Methods section. **A** Representative Western blotting image of wild-type A549 cells and cells with PICT1 deletion. **B** Cells were treated with 20% cigarette smoke extract (CSE) for 24 h. Histograms were obtained by flow cytometry analysis using a DCF-DA probe. **C** DCF fluorescence intensity was quantified. **D** Representative images of comets (scale bar—5 µm). **E** Olive tail moments were quantified. Panel II: Wild-type A549 cells and cells with PICT1 deletion were treated with 20% CSE for 24 h. **A** Representative Western blotting images of MRE11, 53BP1, LIGASE IV, and KU80 expression. **B**-**E** Quantification of protein levels is shown. Panel III: mRNA expression of *MRE11* (A), *53BP1* (B), *KU80* (C), and *LIGASE IV* (D) by RT-PCR. Data are shown as means ± SEM (C – control; *N* = 3 – 9 experimental replicates). *p < 0.05, **p < 0.01, ***p < 0.001, ****p < 0.0001

A549 cell line with PICT1 knockout was generated using the CRISPR-Cas9 strategy, and PICT1 deficiency was confirmed by Western blotting (Fig. 4, Panel I, A). Increased ROS generation in wild-type A549 cells and PICT1-deficient cells compared to controls was detected after exposure to 20% cigarette smoke extract for 24 h (Figs. 4, Panel I, B, C). The highest ROS levels were found in A549 cells with PICT1 knockout. Next, we wanted to define whether PICT1 has a role in DNA damage repair. Wild-type A549 cells and cells with PICT1 deletion were treated with cigarette smoke extract. DNA damage was the highest in PICT1-knockout cells after this treatment compared to all tested conditions (Fig. 4, Panel I, D, E). Furthermore, we analyzed the expression of proteins involved in double-strand breaks (DSB) repair in A549 cells with PICT1 deletion treated with cigarette smoke extract (Fig. 4, Panel II, A). This treatment decreased MRE11 levels in wild-type A549 cells and cells with PICT1 deficiency, and its lowest expression was observed in the latter (Fig. 4, Panel II, B). 53BP1 levels were reduced in A549 cells with PICT1 deficiency (Fig. 4, Panel II, C). However, exposure to cigarette smoke extract increased 53BP1 expression in wild-type A549 cells compared to controls. LIGASE IV and KU80 levels were decreased in PICT1-deficient cells compared to control wild-type A549 cells (Fig. 4, Panel II, D, E). *MRE11* mRNA expression was unaffected by cigarette smoke extract exposure in wild-type and PICT1-deficient A549 cells (Fig. 4, Panel III, A). *53BP1* mRNA levels were reduced in cells with PICT1 knockout treated with cigarette smoke extract compared to controls (Fig. 4, Panel III, B). There was no significant difference in *KU80* expression in all analyzed conditions (Fig. 4, Panel III, C). *LIGASE IV* levels were higher in cells with PICT1 deletion than in control wild-type A549 cells (Fig. 4, Panel III, D). Our data suggest that PICT1 deficiency impairs DSB repair proteins, which can contribute to increased DNA damage.

### The role of PICT1 in replication stress

**Fig. 5:**
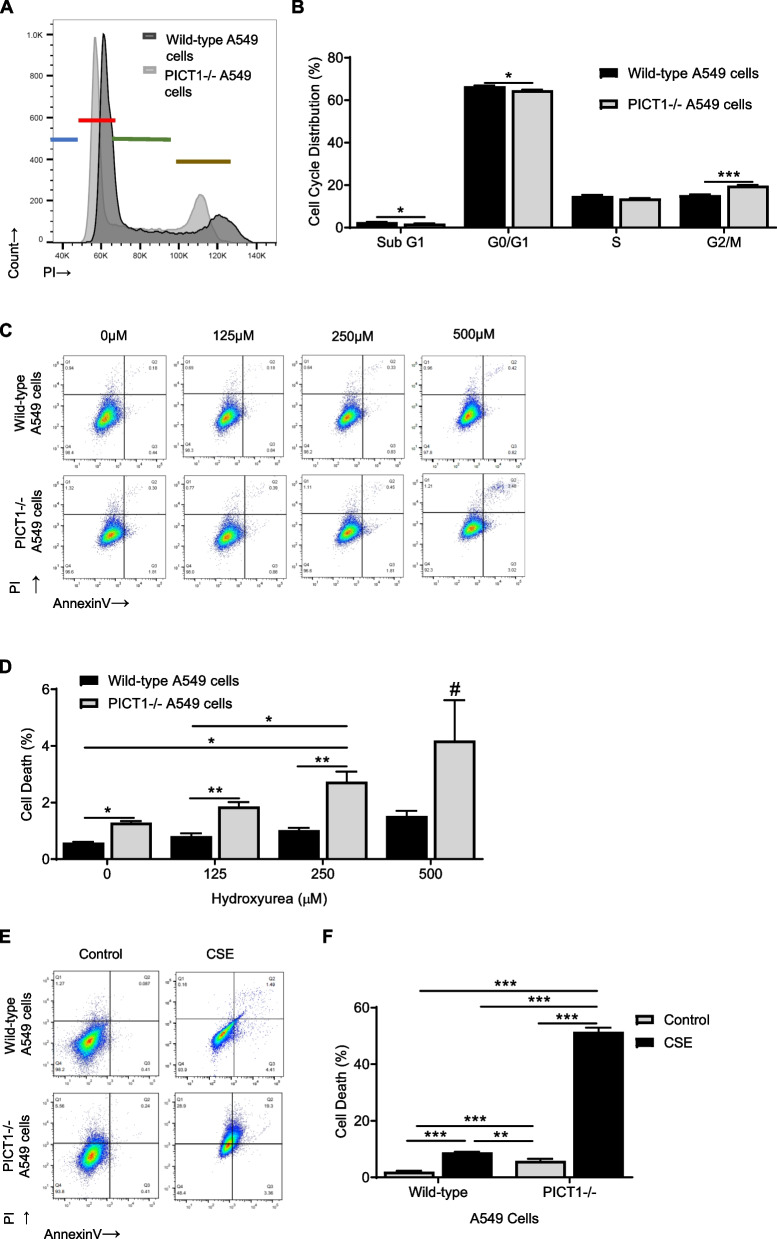
PICT1 deletion sensitizes A549 cells to replicative stress and cell death. **A** Representative cell cycle histograms showing sub-G1 (blue), G0/G1 (red), S (green), and G2/M (brown) phases in wild-type A549 cells and cells with PICT1 deletion using propidium iodide (PI) staining and flow cytometry analysis. **B** Quantification is also shown. **C** Representative flow cytometry plots of wild-type A549 cells and cells with PICT1 deletion treated with hydroxyurea for 24 h and stained using PI and Annexin V. **D** Cell death quantification. **E** Representative flow cytometry plots of wild-type A549 cells and cells with PICT1 deletion treated with 20% cigarette smoke extract (CSE) for 24 h. **F** Cell death quantification is shown. Data represent means ± SEM (*N* = 3 – 9 experimental replicates). *p < 0.05, **p < 0.01, ***p < 0.001, #*p* < 0.001 compared to all tested conditions

A decrease in the subG1 and G0/G1 phases in PICT1 knockout cells was detected compared to wild-type A549 cells (Figs. 5A, B). The number of cells in the G2/M phase of the cell cycle was increased. Cell death increased with the concentration of hydroxyurea, which was enhanced by PICT1 deficiency (Figs. 5C, D). Also, our data indicate that PICT1 knockout led to the highest cell death after treatment with cigarette smoke extract compared to all analyzed conditions (Fig. 5E, F). Our results suggest that PICT1 deficiency sensitizes cells to replication stress and may contribute to cell death.

### PICT1 deletion contributes to mitochondrial dysfunction

**Fig. 6:**
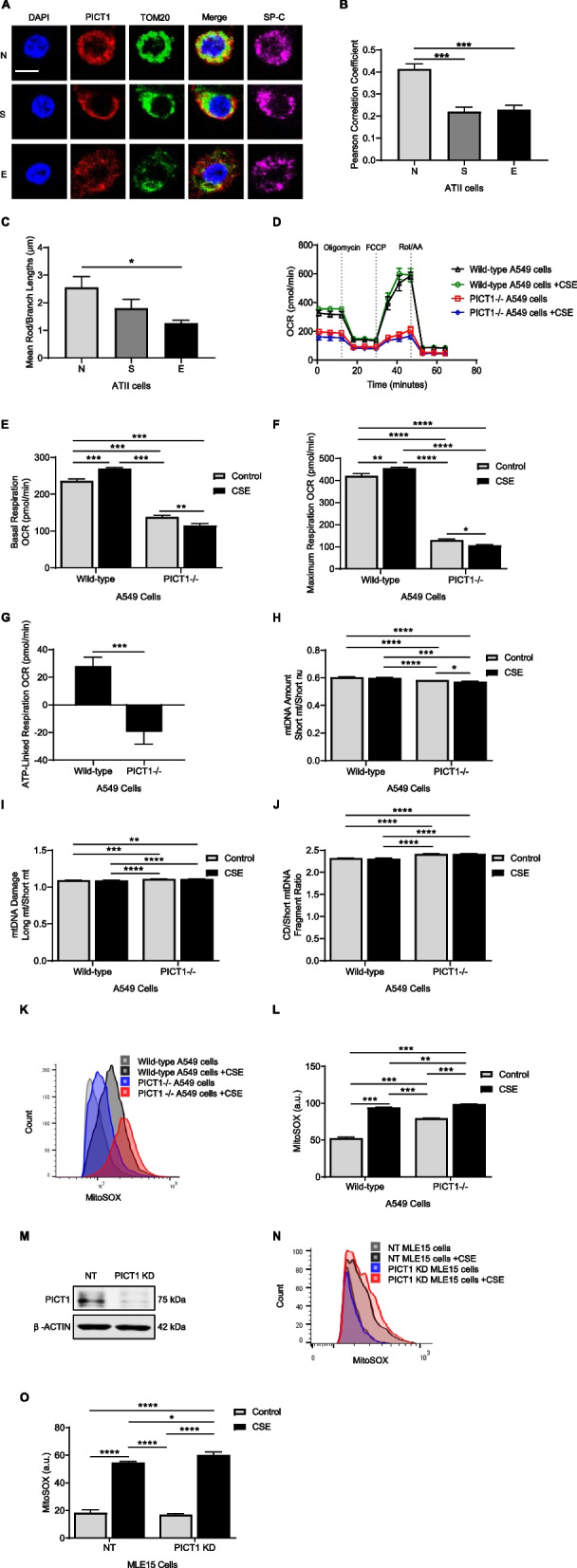
Mitochondrial dysfunction in human primary ATII cells, A549 cells, and MLE15 cells. **A** PICT1 (red), TOM20 (green), and DAPI (blue) staining in lung tissue sections obtained from non-smokers (N), smokers (S), and emphysema patients (E) by immunofluorescence (scale bar—5 μm). ATII cells were identified using SP-C (magenta). **B** Pearson’s correlation coefficient for PICT1 and TOM20 co-localization in ATII cells is shown (*N* = 3 lungs per group). **C** Quantification of mitochondrial networks in ATII cells. **D** Mitochondrial respiration analysis in wild-type A549 cells and cells with PICT1 deletion treated with 20% cigarette smoke extract (CSE) for 24 h. Quantification of basal respiration (**E**) and maximum respiration (**F**) in A549 cells. **G** ATP-linked respiration after exposure to CSE relative to controls in A549 cells. QPCR was used to determine mtDNA amount (**H**), mtDNA damage (**I**), and common deletions (CD, **J**) in A549 cells. Representative histograms using MitoSOX staining and flow cytometry analysis (**K**) and the quantification of fluorescence intensity (**L**) in A549 cells. **M** Representative Western blotting images of MLE15 cells treated with NT (non-target) or PICT1 siRNA. **N** Histograms of MitoSOX staining by flow cytometry analysis (**N**) and quantification (**O**) in MLE15 cells. Data are shown as means ± SEM (KD – knockdown, *N* = 3 – 10 experimental replicates). *p < 0.05, **p < 0.01, ***p < 0.001, ****p < 0.0001

We found reduced mitochondrial co-localization of PICT1 and TOM20 in ATII cells in smokers and emphysema patients compared to non-smokers (Figs. 6A, B). Moreover, we detected a decrease in the mitochondrial network in ATII cells in emphysema patients than in non-smokers (Fig. 6C). The role of PICT1 in mitochondrial metabolism was determined in A549 cells with PICT1 deletion treated with 20% cigarette smoke extract for 24 h. Changes in mitochondrial oxygen consumption rates (OCR) were monitored (Fig. 6D). OCR values were corrected for non-mitochondrial respiration following the addition of the inhibitors rotenone and antimycin A. After adding the ATP synthase inhibitor, oligomycin, the OCR decreased in cells with PICT1 deletion compared to wild-type A549 cells, suggesting ATP-linked respiration was inhibited*.* Moreover, adding the protonophore, FCCP stimulated the OCR to its maximal level in both cell lines but to a lower extent in PICT1-deficient cells. Basal respiration was significantly reduced in PICT1 knockout cells compared to wild-type A549 cells (Fig. 6E). Also, its levels were the lowest in cells with PICT1 deletion treated with cigarette smoke extract compared to all analyzed conditions. This suggests decreased mitochondrial metabolism caused by PICT1 deficiency. Treatment with cigarette smoke extract significantly increased maximum respiration in wild-type A549 cells and reduced it in PICT1 knockout cells compared to controls (Fig. 6F). This can result in lower spare capacity and ATP-coupled respiration in cells with PICT1 deletion. Also, the lowest ATP-linked respiration was observed in PICT1-deficient cells treated with cigarette smoke extract, suggesting general cell bioenergetic deficiency (Fig. 6G). Our results indicate that the mitochondrial oxidative phosphorylation (OXPHOS) system is impaired with loss of PICT1.

MtDNA amount was the lowest in A549 cells with PICT1 knockout treated with cigarette smoke extract compared to all tested conditions (Fig. 6H). MtDNA damage and common deletion (CD) were increased in cells with PICT1 knockout compared to wild-type A549 cells (Figs. 6I, J). Mitochondrial ROS generation was the highest in A549 cells with PICT1 deficiency treated with cigarette smoke extract (Figs. 6K, L). We also analyzed mitochondrial ROS in MLE15 cells with PICT1 knockdown (Fig. 6M, Supplementary Fig. 4). It was the highest in cells with PICT1 knockdown treated with cigarette smoke extract compared to all tested conditions (Figs. 6N, O). Our results indicate that PICT1 deficiency inhibited mitochondrial respiration, increased mitochondrial ROS production, and caused mitochondrial DNA damage, leading to mitochondrial dysfunction.

## Discussion

The role of nuclear-encoded PICT1 in maintaining mitochondrial genome stability highlights the importance of nuclear-mitochondrial interactions. Here, we investigated the impact of PICT1 on mitochondrial function and DNA damage in ATII cells in emphysema. Our results indicate that PICT1 protein expression is significantly decreased in this disease. We also detected its interaction with E3 ubiquitin ligase, TRIM22. To further define PICT1 function in this disease progression, we generated a murine model of emphysema by exposing mice to cigarette smoke for 8 months. The analysis of the structural changes in the lungs by histology and micro-CT confirmed the emphysema phenotype. We observed a gradual decrease in PICT1 protein levels in ATII cells in mice exposed to cigarette smoke for 3 weeks and 8 months. Interestingly, it was reported that PICT1 cytoplasmic expression was significantly higher in smokers with non-small cell lung cancer and correlated with tumor progression and a poor prognostic factor [14].

Our previously published data indicate that emphysema is associated with DNA damage and ATII cell death [8]. Its development is also correlated with inefficient DNA repair capacity [23]. Here, we found that cells with PICT1 knockout exposed to cigarette smoke extract have the highest DNA damage. This can trigger cell cycle arrest and death [24]. Cell cycle checkpoints can slow down or arrest cell cycle progression, allowing the cell to repair or prevent the transmission of damaged chromosomes [25, 26]. Checkpoint machinery can initiate pathways leading to apoptosis and removing damaged cells. Blocking the G2/M transition protects against the segregation of incompletely replicated DNA [27]. Our results indicate that cells with PICT1 knockout have increased the G2/M phase, which suggests the critical role of PICT1 in maintaining genome stability. It was shown that *Pict1* loss led to enhanced apoptosis in embryonic stem cells [13]. Moreover, *Pict1* KO pups were embryonic lethal, which indicates its crucial function.

MtDNA damage is linked to OXPHOS impairment and is associated with respiratory diseases that may result from DNA repair defects and mitochondrial dysfunction [28]. Here, we detected a link between PICT1 deficiency and mitochondrial abnormalities, including increased ROS, DNA damage, and altered metabolism, which can contribute to cell death. Reports have shown that rare respiratory diseases, such as alpha-1 antitrypsin deficiency (AATD), which can lead to emphysema, are associated with high oxidative stress and mitochondrial dysfunction [29–31]. Further studies are needed to determine the link between PICT1 function and AATD. We demonstrate that ATII cells isolated from emphysema patients have reduced PICT1 expression and impaired mitochondrial function. We reported an increase in MitoSOX in this disease [10]. Also, cells with PICT1 deficiency had the highest cell death, indicating inefficient DNA damage repair. Our data suggests that PICT1 is a novel factor contributing to nuclear DSB repair and mitochondrial function, and the impairment of these processes may contribute to emphysema development. We found that PICT1 and MRE11 interaction was decreased in smokers and emphysema patients, in mice exposed to cigarette smoke for 3 weeks, and in the murine model of this disease. Also, reduced MRE11 and PICT1 levels in human and murine emphysema suggest DSB repair defects, which can lead to impaired formation of the MRN complex. The lowest MRE11 protein expression was detected in cells with PICT1 knockout and correlated with decreased 53BP1, LIGASE IV, and KU80 levels. It was reported that MRE11 is essential in repairing DSBs, which are the most cytotoxic DNA lesions [32]. High MRE11 levels were observed in the cytoplasm in peripheral blood lymphocyte and airway epithelial cells in individuals exposed to biomass smoke [33]. However, MRE11 activity was inhibited while it moved from the nucleus to the cytoplasm, and cells became more susceptible to DNA damage. MtDNA is vulnerable to oxidative damage induced by exogenous and endogenous ROS due to its proximity to the ETC and the lack of protective histones [34]. Our results indicate the highest mtDNA damage and the lowest mtDNA amount in ATII cells in emphysema compared to control non-smokers and smokers [10]. Here, we found mtDNA damage, common deletions, and decreased mtDNA amount in cells with PICT1 knockout exposed to cigarette smoke extract. Common deletion is located between nucleotides 8,470 and 13,459 and can contribute to mitochondrial dysfunction [35]. Our results show mitochondrial respiration inhibition in cells with PICT1 deficiency. This suggests that PICT1 can constitute a significant component of mitochondrial signaling, contribute to maintaining mitochondrial genome stability, and highlight the importance of nuclear-mitochondrial interactions. Our results are in agreement with data showing that PICT1 enhances mitochondrial function and is required to maintain oxygen consumption, consistent with its critical role in controlling cellular respiration [12].

## Conclusions

**Fig. 7:**
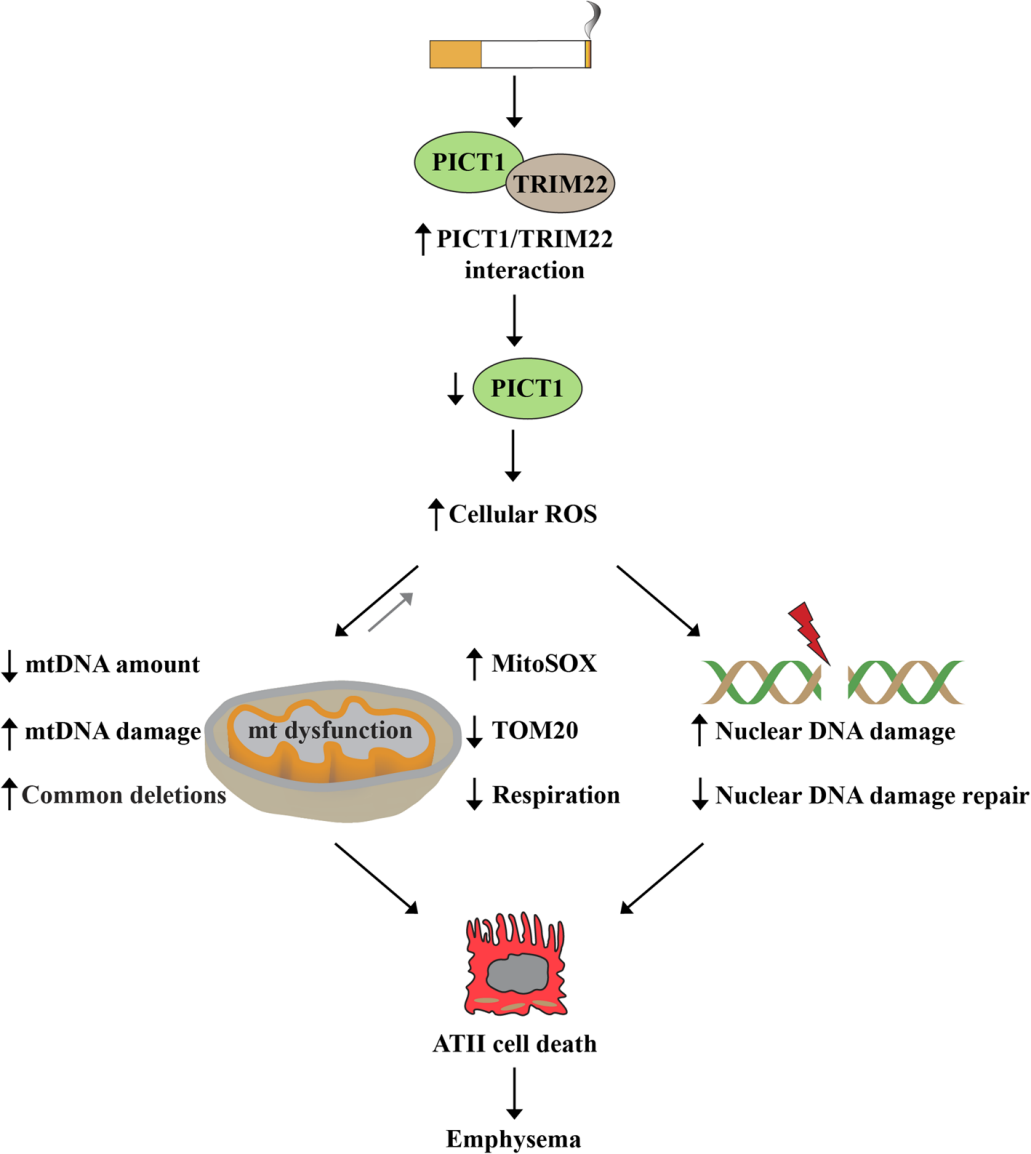
The role of PICT1 in nuclear DNA damage and mitochondrial (mt) function. Increased PICT1/TRIM22 interaction induced by smoking leads to decreased PICT1 levels and high ROS production. This caused nuclear and mtDNA damage, common deletions, mitochondrial superoxide generation, and reduced mtDNA amount and respiration, contributing to ATII cell death and emphysema development

Our results indicate that PICT1 deficiency contributes to mitochondrial dysfunction, which is attributable to increased ROS and nuclear and mtDNA damage, contributing to cell death and emphysema (Fig. 7). Therapeutic approaches to stabilizing and restoring PICT1 function may lead to potential clinical strategies for treating emphysema.

## Supplementary Information

Supplementary Material 1.

Supplementary Material 2.

## Data Availability

All data generated or analyzed during this study are included.

## Abbreviations

ATII cells: Alveolar type II cells
COPD: Chronic obstructive pulmonary disease
DSB: Double strand breaks
Mt: Mitochondrial
OXPHOS: Mitochondrial oxidative phosphorylation
MOM staining kit: Mouse on mouse staining kit
MRN: MRE11-RAD50-NBS1
MS/MS spectra: Tandem mass spectrometry
PI: Propidium iodide
PICT1: Protein interacting with carboxyl terminus-1
ROS: Reactive Oxygen Species
TSP: Total suspended particles
