# Supplementary material for: Mitochondrial dysfunction and impaired DNA damage repair through PICT1 dysregulation in alveolar type II cells in emphysema
Source: Cell Commun Signal. 2024 Nov 22;22:562. doi: 10.1186/s12964-024-01896-0 (PMC11583753; doi:10.1186/s12964-024-01896-0)
Supplement: Supplementary file 1 — Supplementary Material 1. [file 12964_2024_1896_MOESM1_ESM.pdf]

SUPPLEMENTARY MATERIALS AND METHODS

Mitochondrial dysfunction and impaired DNA damage repair through PICT1 dysregulation in alveolar type II cells in emphysema

Hannah Simborio[1], Hassan Hayek[1,2], Beata Kosmider[1,2], John W. Elrod[4], Sudhir Bolla[3], Nathaniel Marchetti[3], Gerard Criner[3], Karim Bahmed[1,2 *]

[1]Center for Inflammation and Lung Research, Lewis Katz School of Medicine at Temple University, Philadelphia, PA 19140, USA

[2]Department of Microbiology, Immunology, and Inflammation, Lewis Katz School of Medicine at Temple University, Philadelphia, PA 19140, USA

[3]Aging & Cardiovascular Discovery Center, Lewis Katz School of Medicine at Temple University, Philadelphia, PA 19140, USA

[4]Department of Thoracic Medicine and Surgery, Lewis Katz School of Medicine at Temple University, Philadelphia, PA 19140, USA

*Corresponding author: Karim Bahmed, Ph.D.
Center for Inflammation and Lung Research
Department of Microbiology, Immunology, and Inflammation
Temple University
3500 N. Broad Street, Philadelphia, PA 19140
E-mail: karim.bahmed@temple.edu

Human ATII cell isolation

Lung tissue was obtained from non-smoker and smoker organ donors whose lungs were unsuitable for transplantation and donated by the Gift of Life Foundation (Philadelphia, PA). We also got lungs from transplants of emphysema patients through Temple University Hospital. ATII cells were isolated, as we previously reported (1). Briefly, 12.9 U/ml elastase (GoldBio) was instilled into the lung, followed by tissue mincing and centrifugation to collect the cell suspension. The cells were filtrated and purified using Optiprep (Accurate Chemical Scientific Corp.). EpCAM microbeads (Miltenyi Biotec Inc.) were used for a positive selection of ATII cells. Freshly isolated ATII cells were used for all experiments.

Human and murine lung fractions

Lung tissue was minced into small pieces and homogenized using SubCell Buffer-I and II (G-Biosciences) according to the manufacturer's protocol. Briefly, the homogenate was centrifuged at 700xg for 5 min to pellet nuclei. The supernatant was centrifuged at 12,000xg for 10 min. The resulting pellet contained mitochondria, while the supernatant was collected as a cytoplasmic fraction.

Chest CT scans

Lung tissue was obtained from areas with mild and severe emphysema from the same patient. Emphysema was defined by the percent of the lung voxels on an inspiratory CT scan with attenuation < -950 HU (2). It was considered absent in subjects with values for Insp−950 < 4% in smokers since increased lung density results in a decrease in emphysema index (3). Severe emphysema was defined by Insp−950 > 14% in smokers. Briefly, the lungs were removed from

the thorax, inflated with air, and frozen using liquid nitrogen vapor, followed by processing into 2-cm thick slices in the same plane as the CT scan. Lung tissue cores were obtained using a sharpened steel cylinder diameter of 1 cm, as we previously described (4). Subjects provided written informed consent for using lung tissue and clinical and radiological data for research.

PICT1 knockout in A549 cells

A549 cells were transfected with PICT1 CRISPR plasmid (Santa Cruz Biotechnology) for 24h and sorted for GFP-positive clones using BD Influx cell sorter (BD Biosciences). A selected A549 clone with PICT1 deletion was validated by Western blotting using a PICT1 antibody (Santa Cruz Biotechnology). Wild-type A549 and PICT1 knockout cell lines were exposed to 20% cigarette smoke extract. Briefly, we used one 3R4F cigarette with no filter (Kentucky Tobacco Research and Development Center). The extract was prepared in 12.5 ml of Dulbecco's modified Eagle's medium without FBS using a peristaltic pump (Mannostat, 72-310-000, Thermo-Fisher Scientific). Cells were collected after 24h for downstream assays.

Comet assay

OxiSelect Comet Assay (Cell Biolabs) was used according to the manufacturer's instructions. Briefly, cells were mixed with LMP agarose and incubated with lysis and alkaline buffers. Fragmented and intact DNA were separated for 30 min at 0.73 V/cm on slides in a horizontal electrophoresis chamber. Slides were dried, and DNA was stained. Pictures were captured using fluorescence microscopy (Zeiss Axioskop 2) and analyzed using OpenComet software (5). DNA damage was quantified as we previously described (6).

Flow cytometry analysis

Alexa Fluor 488 Annexin V/Dead Cell Apoptosis kit (Thermo Fisher Scientific) was applied per the manufacturer's instructions. Cells were stained in a binding buffer containing 5 μL Annexin V conjugated to Alexa Fluor 488 and 1 μg/mL propidium iodide (PI) for 5 min. LSR-II flow cytometer (BD Biosciences) and FlowJo (TreeStar) were used for data acquisition and analysis. Briefly, 1×10^5 cells were incubated with 10 μM DCF-DA for 45 min at 37 °C, and the analysis was performed using an LSR-II flow cytometer, as we previously reported (6).

Mitochondrial function

Genomic DNA was isolated from wild-type A549 and PICT1 deficient cells using EasyPrep Genomic DNA Kit (Bioland Scientific) to determine mitochondrial DNA (mtDNA) amount, mtDNA damage, and common deletion. Mitochondrial metabolism was measured using the Seahorse XF technology (Agilent). Briefly, cells were seeded in Seahorse XF plates and cultured for 24h prior to assay. Basal and maximum mitochondrial respiration and ATP-linked respiration were analyzed using Seahorse XF96 Extracellular Flux Analyzer (Seahorse Bioscience, Agilent).

Table S1. Sequences of human primers used for RT-PCR.

Target	Sequences
53BP1	Fw: ATG GAC CCT ACT GGA AGT CAG<br>Rev: TTT CTT TGT GCG TCT GGA GAT T
GAPDH	Fw: GGA GCG AGA TCC CTC CAA AAT<br>Rev: GGC TGT TGT CAT ACT TCT CAT GG

KU80	Fw: GCA CTG ACA ATC CCC TTT CTG<br>Rev: TCA ATG TCC TCC AGC AAA TCA AA
LIGASE IV	Fw: AGC AAA AGT GGC TTA TAC GGA TG<br>Rev: TGA GTC CTA CAG AAG GAT CAT GC
MRE11	Fw: ATG CAG TCA GAG GAA ATG ATA CG<br>Rev: CAG GCC GAT CAC CCA TAC AAT
PICT1	Fw: GTA CCA GGC ACC TGA CAT CG<br>Rev: ACC TTG TAC TTG CGT TTG AAC T
TRIM22	Fw: CTG TCC TGT GTG TCA GAC CAG<br>Rev: TGT GGG CTC ATC TTG ACC TCT

Table S2. Sequences of murine primers used for RT-PCR.

Target	Sequences
Gapdh	Fw: CAT GGC CTT CCG TGT TCC T<br>Rev: CCT GCT TCA CCA CCT TCT T
Mre11	Fw: CCT CTT ATC CGA CTA CGG GTG<br>Rev: ACT GCT TTA CGA GGT CTT CTA CT
Pict1	Fw: CGA AAG GAG GAG TTA TGG GAG A<br>Rev: TCA TAG AAG GGT CGC TCA ATG AT

REFERENCES

1. Kosmider B, Mason RJ, Bahmed K. Isolation and Characterization of Human Alveolar Type II Cells. Methods Mol Biol. 2018;1809:83-90.

2. Gevenois PA, De Vuyst P, de Maertelaer V, Zanen J, Jacobovitz D, Cosio MG, et al. Comparison of computed density and microscopic morphometry in pulmonary emphysema. Am J Respir Crit Care Med. 1996;154(1):187-92.

3. Shaker SB, Stavngaard T, Laursen LC, Stoel BC, Dirksen A. Rapid fall in lung density following smoking cessation in COPD. COPD. 2011;8(1):2-7.

4. Lin CR, Bahmed K, Criner GJ, Marchetti N, Tuder RM, Kelsen S, et al. S100A8 Protects Human Primary Alveolar Type II Cells against Injury and Emphysema. Am J Respir Cell Mol Biol. 2019;60(3):299-307.

5. Gyori BM, Venkatachalam G, Thiagarajan PS, Hsu D, Clement MV. OpenComet: an automated tool for comet assay image analysis. Redox Biol. 2014;2:457-65.

6. Kosmider B, Lin CR, Vlasenko L, Marchetti N, Bolla S, Criner GJ, et al. Impaired non-homologous end joining in human primary alveolar type II cells in emphysema. Sci Rep. 2019;9(1):920.
