# Supplementary material for: Mitochondrial dysfunction and impaired DNA damage repair through PICT1 dysregulation in alveolar type II cells in emphysema
Source: Cell Commun Signal. 2024 Nov 22;22:562. doi: 10.1186/s12964-024-01896-0 (PMC11583753; doi:10.1186/s12964-024-01896-0)
Supplement: Supplementary file 2 — Supplementary Material 2. [file 12964_2024_1896_MOESM2_ESM.pdf]

SUPPLEMENTARY RESULTS

Mitochondrial dysfunction and impaired DNA damage repair through PICT1 dysregulation in alveolar type II cells in emphysema

Hannah Simborio[1], Hassan Hayek[1,2], Beata Kosmider[1,2], John W. Elrod[4], Sudhir Bolla[3], Nathaniel Marchetti[3], Gerard Criner[3], Karim Bahmed[1,2] *

[1]Center for Inflammation and Lung Research, Lewis Katz School of Medicine at Temple University, Philadelphia, PA 19140, USA

[2]Department of Microbiology, Immunology, and Inflammation, Lewis Katz School of Medicine at Temple University, Philadelphia, PA 19140, USA

[3]Aging & Cardiovascular Discovery Center, Lewis Katz School of Medicine at Temple University, Philadelphia, PA 19140, USA

[4]Department of Thoracic Medicine and Surgery, Lewis Katz School of Medicine at Temple University, Philadelphia, PA 19140, USA

*Corresponding author: Karim Bahmed, Ph.D.
Center for Inflammation and Lung Research
Department of Microbiology, Immunology, and Inflammation
Temple University
3500 N. Broad Street, Philadelphia, PA 19140
E-mail: karim.bahmed@temple.edu

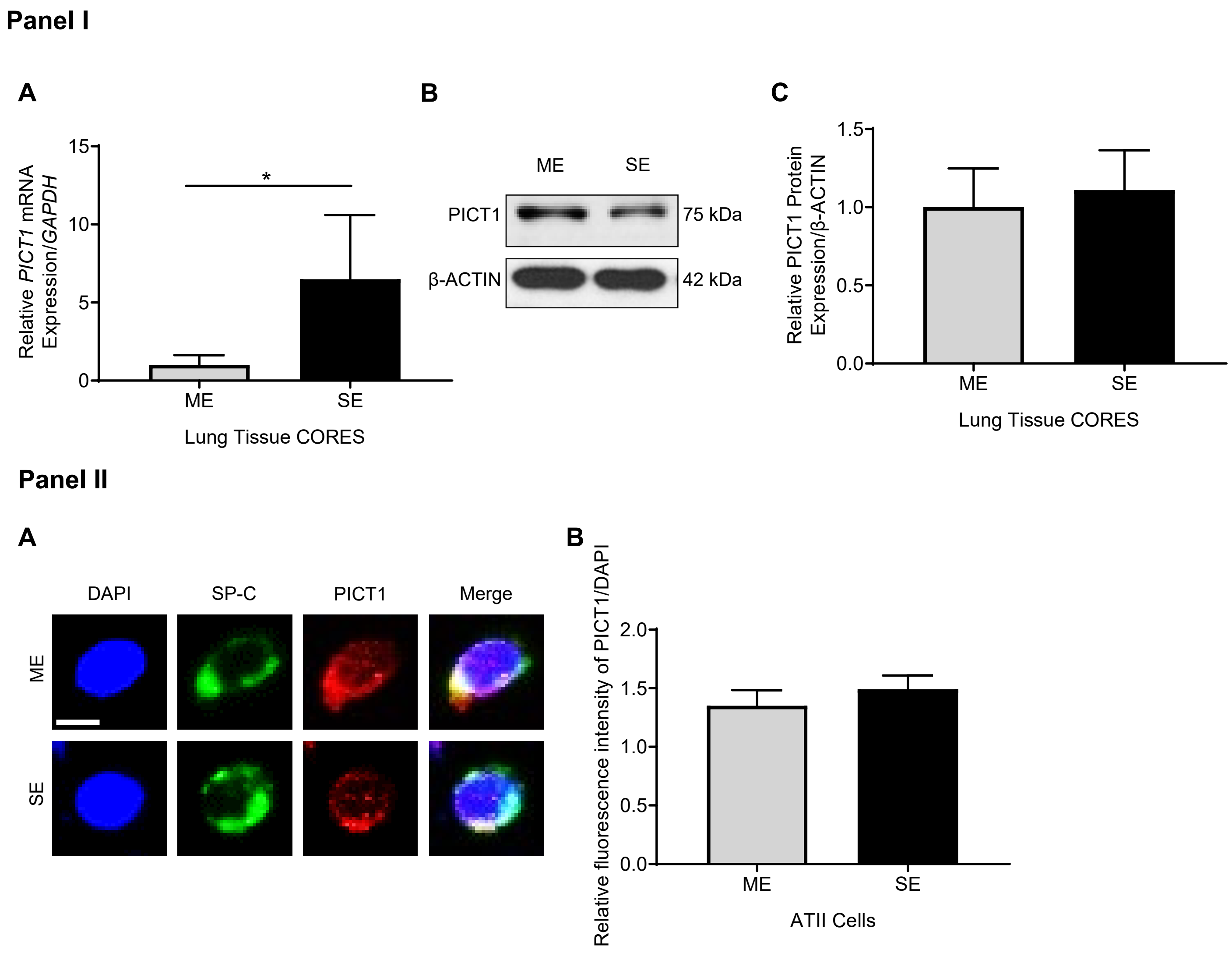

Figure S1. PICT1 expression in mild and severe emphysema. Panel I: A - *PICT1* mRNA levels in lung tissue cores obtained from areas with mild and severe emphysema of the same patient by RT-PCR (ME – mild emphysema; SE – severe emphysema). B - Representative Western blotting images of PICT1 expression. C - Quantification of PICT1 levels is shown. Panel II: A – PICT1 (red) expression in ATII cells identified in lung tissue sections using SP-C (green) and DAPI (blue; scale bar - 5 μm) by immunofluorescence. B - Quantification of PICT1 fluorescence intensity. Data are shown as means ± SEM (N = 3 – 7 lungs per group). *$p<0.05$.

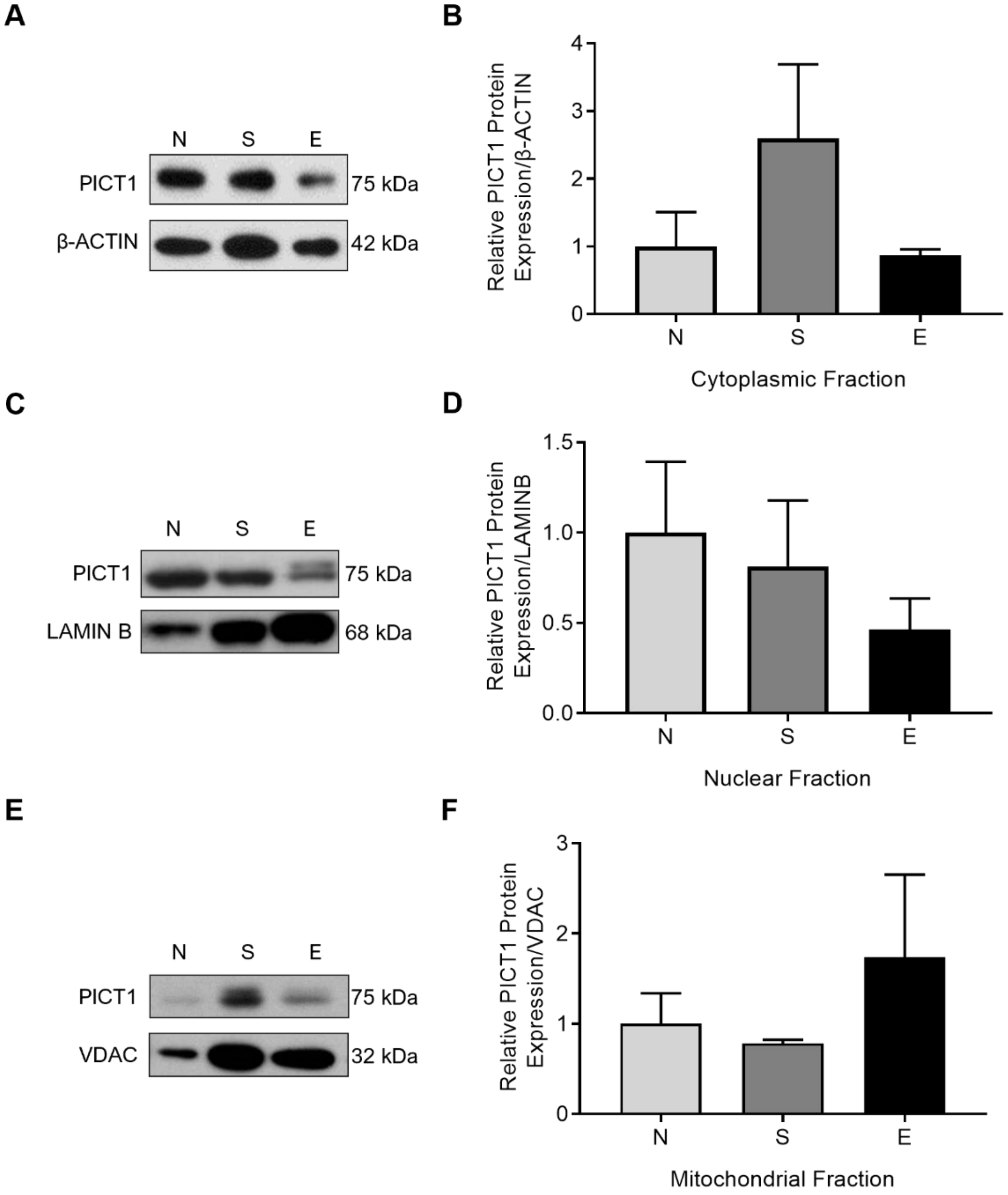

Figure S2. Expression of PICT1 in human lung tissue fractions. Lung tissue was obtained from non-smokers (N), smokers (S), and emphysema patients (E). PICT1 expression was analyzed in cytoplasmic (A, B), nuclear (C, D), and mitochondrial (E, F) fractions. Representative Western blotting images and quantifications are shown. Data are shown as means ± SEM (N = 3 lungs per group).

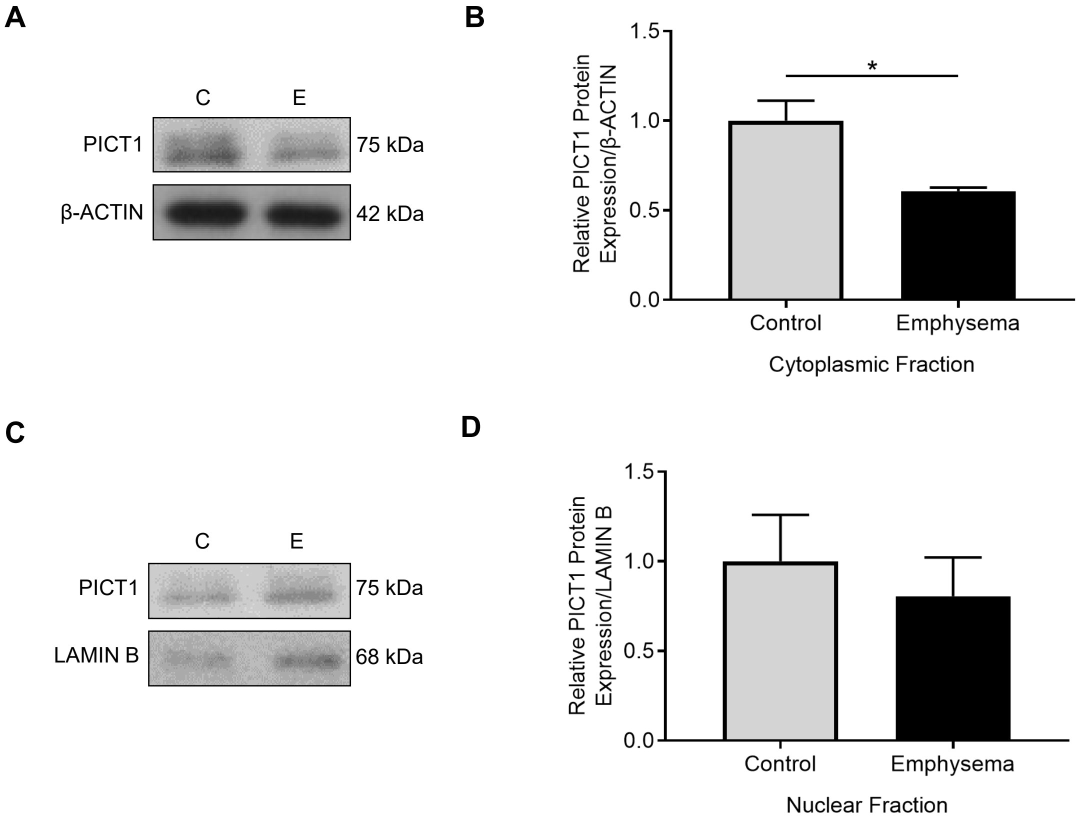

Figure S3. Decreased PICT1 protein levels in cytoplasmic fractions in the lung tissue in murine emphysema. Wild-type mice were exposed to cigarette smoke for 8 months, as described in the Methods section, to induce emphysema. PICT1 expression was analyzed in cytoplasmic (A, B) and nuclear (C, D) fractions. Representative Western blotting images and quantifications of protein levels are shown. Data are shown as means ± SEM (N = 3 mice per group). $*p<0.05$.

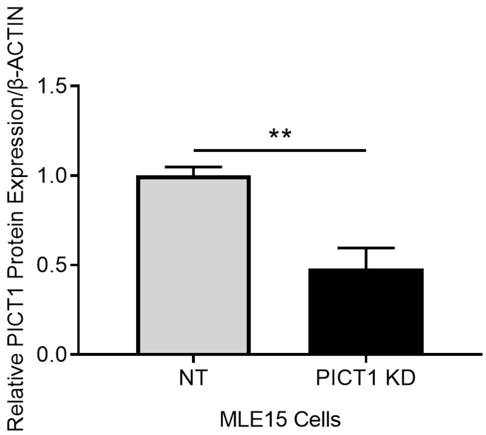

Figure S4. PICT1 knockdown in MLE15 cells. siRNA strategy was used to knock down PICT1. Quantification of PICT1 protein levels is shown (NT- Non-target siRNA, KD – knockdown). Data are shown as means ± SEM (N = 3 replicates). $^{**}p<0.01$.
